# Factors influencing psychosocial adjustment and quality of life in Parkinson patients and informal caregivers

**DOI:** 10.1007/s11136-015-1220-3

**Published:** 2016-01-08

**Authors:** María Victoria Navarta-Sánchez, Juana M. Senosiain García, Mario Riverol, María Eugenia Ursúa Sesma, Sara Díaz de Cerio Ayesa, Sagrario Anaut Bravo, Neus Caparrós Civera, Mari Carmen Portillo

**Affiliations:** Faculty of Nursing, University of Navarre, Navarre, Spain; Department of Neurology, Clínica Universidad de Navarra, Navarre, Spain; Primary Health Care Center of San Juan, Navarre Service of Health-Osasunbidea, Navarre, Spain; Navarre Association of Parkinson’s Patients, Navarre, Spain; Department of Social Work, Public University of Navarre, Navarre, Spain; Faculty of Law and Social Sciences, University of La Rioja, La Rioja, Spain; Faculty of Health Sciences, University of Southampton, Highfield, Southampton, SO171BJ UK

**Keywords:** Quality of life, Parkinson’s disease, Psychosocial variables, Caregivers

## Abstract

**Objective:**

The influence that social conditions and personal attitudes may have on the quality of life (QoL) of Parkinson’s disease (PD) patients and informal caregivers does not receive enough attention in health care, as a result of it not being clearly identified, especially in informal caregivers. The aim of this study was to provide a comprehensive analysis of psychosocial adjustment and QoL determinants in PD patients and informal caregivers.

**Methods:**

Ninety-one PD patients and 83 caregivers participated in the study. Multiple regression analyses were performed including benefit finding, coping, disease severity and socio-demographic factors, in order to determine how these aspects influence the psychosocial adjustment and QoL in PD patients and caregivers.

**Results:**

Regression models showed that severity of PD was the main predictor of psychosocial adjustment and QoL in patients. Nevertheless, multiple regression analyses also revealed that coping was a significant predictor of psychosocial adjustment in patients and caregivers. Furthermore, psychosocial adjustment was significantly related to QoL in patients and caregivers. Also, coping and benefit finding were predictors of QoL in caregivers but not in patients.

**Conclusions:**

Multidisciplinary interventions aimed at improving PD patients’ QoL may have more effective outcomes if education about coping skills, and how these can help towards a positive psychosocial adjustment to illness, were included, and targeted not only at patients, but also at informal caregivers.

## Introduction

Nowadays, advances in pharmacology and surgical treatments may control or reduce motor and non-motor symptoms in Parkinson’s disease (PD) [1]. However, positive outcomes of these therapies differ among patients and even decline over time due to the chronic and neurodegenerative character of PD. Furthermore, most non-pharmacological interventions to improve how people live with PD are focused on self-management [2]. Nevertheless, patients do not receive education to encourage their awareness about their health as a dynamic state, which is influenced by their chronic illness and personal circumstances. In addition, current health care does not seem to promote the patients’ psychosocial adjustment to illness, although this is a relevant process in their health illness cycle [3, 4].

For PD patients, the care their informal caregivers provide them with is essential, due to their progressive dependence and disability. However, this informal care may also have a negative impact on the caregiver’s health and QoL [5–7]. Furthermore, informal caregivers confront multiple challenges which require a significant effort for psychosocial adjustment [4, 8–10].

At present, research to determine the relationship between psychosocial adjustment to PD and QoL in PD patients and caregivers is limited. However, the first study which explored this relationship showed that psychosocial adjustment to PD has a significant influence on QoL [11]. Helping PD patients and caregivers to achieve positive adjustment to the illness is missing, despite the potential impact this may have on their QoL [11]. Furthermore, the definition of main predictors of QoL continues to be unclear in PD patients, but even more so in their caregivers [12–15]. Recent evidence about determinants of QoL suggests that it may be beneficial to consider socio-demographic factors, motor and non-motor symptoms to improve QoL in PD patients and caregivers [12–15].

Qualitative research shows that some PD patients and informal caregivers develop coping strategies to face the difficulties in day-to-day life, such as having a positive attitude and engaging in meaningful activities [16–18]. Moreover, studies in other chronic illnesses state that some patients and informal caregivers may actually perceive positive benefits as a consequence of the illness, for example, personal growth and an improvement in family relationships [19]. These investigations show that coping strategies and benefit finding might facilitate psychosocial adjustment to illness [16–19]. Consequently, there is a need to know the relationships between psychosocial adjustment to PD, coping responses, benefit finding, socio-demographic factors and QoL, if effective interventions to tackle these gaps are to be developed.

The present study aimed to explore potential clinical, social and attitudinal determinants of psychosocial adjustment and QoL in PD patients and informal caregivers. Based on previous studies [4, 8–15] we hypothesized that coping responses, finding benefit in the illness, disease severity, and socio-demographic factors would contribute significantly to psychosocial adjustment, and that all previous factors would also predict QoL in PD patients and informal caregivers.

## Methods

### Study design

A cross-sectional evaluation and multi-center design was implemented after obtaining approval from the Ethics Committee of the University of Navarre. The present study is part of a comprehensive research programme (ReNACE) aimed at designing multidisciplinary education interventions to help patients with chronic illnesses and their family carers to live with a long term condition. The results of this publication were obtained in the first phase of this research.

Patients were recruited through consecutive sampling from three settings in a community context: the Navarre association of Parkinson’s patients, a Neurology outpatient clinic and a primary care practice in Pamplona (Spain). In this way, the recruitment of participants was developed in all possible contexts at the community in Pamplona: voluntary sector, private health institution and public health care system.

Patients were included in the study if they fulfilled the UK PD Society Brain Bank diagnostic criteria for PD according to their neurologists [20], received ambulatory attention in the selected centers and their permanent residence was in Pamplona. Patients were excluded if their neurologists stated that they presented dementia according to the Movement Disorders Society criteria [21]. Eligible informal caregivers were included if they usually cohabited with the patient, were directly responsible for his/her care (although this could include only emotional support) and provided unpaid care following the definition of informal caregiver of Dyck and Calne [22]. Both patients and informal caregivers were included if they could understand and complete the measuring instruments in Spanish. Professionals in the three settings received information about the inclusion and exclusion criteria. All eligible patients and informal caregivers who fulfilled inclusion criteria were informed about the study by professionals in their own consultations. Patients and informal caregivers interested in the study were contacted by the research team to check the inclusion criteria and explain more details about the objectives of this study. The sample size was estimated to fulfill the rule 10 events per predictor variable [23]. Therefore, the minimum sample size estimated was 80 patients and 80 informal caregivers. All patients and caregivers who met the criteria during the recruitment time (7 months) and provided informed consent were included in the study. Thus, this study was carried out with the highest number of possible participants in Pamplona at the time of the study, which was suitable for the four multivariable analyses done [23].

### Measures

The *39*-*item PD Questionnaire* (PDQ-39) was used to rate patients’ health-related QoL [24]. The PDQ-39 comprises 39 items which are divided into 8 areas: mobility, ADL’s, emotional well-being, stigma, social support, cognition, communication and bodily discomfort. Each item is rated on a 1–5-point Likert scale for assessing the frequency in which respondents experienced the problem defined in each item. The scores for each aspect are expressed as a percentage score ranging from 0 (not problem at all) to 100 (maximum level of problem). The summary index is calculated through the total sum of the percentage score of each domain divided by eight. This scale is internationally used and recommended in clinical practice and research [25], and is validated in Spanish [26]. In the current data the consistency reliability was satisfactory (*α* = 0.93).

Caregivers’ QoL was assessed with the *Scale of QoL of Caregivers* (SQLC) [27]. This is a scale of 16 items which measures the impact of the disease on three areas: professional activity, social and leisure activities and responsibilities of the caregiver to help the patient in his/her everyday life. The SQLC has a double system of rating (items and sub-items) and the lower scores indicate a more severe impact on the caregiver´s QoL. The level of impact on QoL is classified in four degrees: none (141–149), mild (100–140), moderate (86–99) and severe (less than 85 points) [27]. The validated Spanish version was used [28]. The internal consistency of SQLC in the study’s caregivers was satisfactory (*α* = 0.86).

The *Psychosocial Adjustment to Illness Scale Self*-*Report* (PAIS-SR) was used to evaluate the psychological and social adjustment of patients and caregivers to the patient´s illness [29]. This multidimensional scale refers to experience in the last 30 days, excluding time in hospital. The PAIS-SR is comprised of 46 items grouped into 7 domains: health care orientation, vocational environment, domestic environment, sexual relationships, extended family relationships, social environment, and psychological distress. Each item is rated on a 0–4 point Likert scale. A global score above 62 points indicates that the person presents difficulties in his/her adjustment to illness. The scale is internationally used in different medical illness [30, 31]. The Self-report version with patients and caregivers was used. The specific version for patients is validated for the Spanish population [32]. However, the PAIS-SR version for caregivers had not been validated in Spanish. Therefore, a process of back-translation with three bilingual people who were experts in psychosocial research and neurological diseases was developed to obtain a Spanish version [33]. In the current data the consistency reliability was satisfactory for patients and caregivers (*α* = 0.89, *α* = 0.87).

The *Brief Cope Scale* was used to measure the different coping responses of PD patients and caregivers [34]. It is a 24-item questionnaire which is classified into 12 scales which refer to different ways of coping to stressful events: active coping, planning, positive reframing, acceptance, humor, religion, using emotional support, using self-distraction, denial, venting, substance use and behavioral disengagement. The score for each item varies from 1 (not doing the action at all) to 4 (doing it very frequently) [34]. High scores indicated a high frequency of coping responses. The scale is validated in Spanish [35]. The Cronbach´s alpha of Brief Cope Scale for patients and caregivers in this study was greater than 0.80.

The *Benefit Finding Scale* was used to measure the finding of benefit as a consequence of PD in patients and caregivers [36]. The scale was developed for women diagnosed with breast cancer, although it has already been applied in studies with patients with different chronic illnesses. The Benefit Finding Scale is a 17-item self-report questionnaire with a 1–5 point Likert scale [36]. High scores indicated greater perception of benefit finding, such as improvement of family and social relationships, a change in life priorities or a reinforcement of a sense of spirituality. The Benefit Finding Scale had not been validated into Spanish. Consequently, a process of back-translation with the three bilingual people mentioned above was developed to obtain a Spanish version [33]. The internal consistency of the scale in the present sample of patients and caregivers was satisfactory (*α* = 0.92, *α* = 0.93).

The *Hoehn and Yahr stage* was used to evaluate the overall severity of the disease in patients with PD [37]. This scale classifies the severity of PD into five stages and is used worldwide.

### Data collection

Participants were helped by a researcher (the first author) to complete the scales during an individual meeting. Most participants (85.6 %) chose face-to-face interviews (at their home or center). Only a few participants (14.4 %) opted to have the interview by telephone because they did not have availability to have a face-to-face appointment. In the telephone interviews, the first author followed the next protocol: introducing herself and the research programme; asking about doubts; reading out loud instructions of the scale; reading out loud each question and its answer options; writing a check mark in the answer chosen by the participant.

### Statistical analyses

Descriptive statistics were applied to determine the socio-demographic and clinical characteristics of the participants. The reliability of scales was assessed using Cronbach´s alpha. Correlations among independent variables in multiple regression analyses were performed using Pearson correlations (with both continuous variables), Phi-coefficient (with two dichotomous variables) and Point bi-serial correlations (a dichotomous variable with a continuous variable). The significance level was set at 0.05.

Multiple linear regression models were adjusted by age and gender. Four models were examined: two models included only variables from patients and two models included only variables from caregivers. In both groups, the first model attempted to determine the major predictors of psychosocial adjustment, and the second model sought to identify the major determinants of QoL. In the case of patients, the first model included the following variables: coping responses, benefit finding, resources (no resources or having one resource versus having two or more resources), Hoenh and Yahr stage (early stages versus advanced stage) and availability of caregiver. The second model added one variable, the psychosocial adjustment. For caregivers, the first model included these variables: coping responses, benefit finding, resources (no resources or having one resource versus having two or more resources), number of years as an informal caregiver and sharing a home with the patient. The second model also added one variable, the psychosocial adjustment. In all models the variables were represented by the total score of the scales of psychosocial adjustment, coping, benefit finding and quality of life. The suitability of the four final models was assessed by the level of significance of each variable, the maximisation of the adjusted determination coefficient (*R*^2^) and the minimisation of the mean squared error (MSE). The independent variables were tested for collinearity.

Statistical analyses were performed with SPSS Version 15.0.

## Results

### Descriptive statistics of the sample

Ninety-one patients and 83 caregivers met the study criteria, provided informed consent and completed scales between June and December 2013. Of these, 87 % (*n* = 76) were patient-caregiver pairs and 22 people participated without their partner. The response rate was 65 % in patients and 55 % in caregivers. The demographic characteristics of the patients and caregivers sample and the scores that they obtained in the scales are presented in Table 1. Regarding resources, participants were asked if they had started to make use of any of the following resources due to PD: Parkinson’s Association, physiotherapy, psychological therapy, social work, primary health care, home care, tele-assistance, part-time nursing homes, financial help or others which are not mentioned. Most of the patients (79.1 %) and half of the caregivers (54.2 %) indicated that they used more than two of the resources stated above.

**Table 1:** Demographic and clinical characteristics of the sample

	Patients (*n* = 91)	Caregivers (*n* = 83)
Gender, *n* (%)		
Male	52 (57.1)	18 (21.7)
Age (years)		
Range	38–93	27–90
Mean (SD)	71.9 (9.5)	63 (13.9)
Marital status, *n* (%)		
Married	60 (65.9)	66 (79.5)
Education, *n* (%)		
Elemental studies	44 (48.4)	32 (38.6)
Employment status, *n* (%)		
Retired	64 (70.3)	29 (34.9)
Full-time job	2 (2.2)	19 (22.9)
Housework	17 (18.7)	21 (25.3)
Hoehn and Yahr, *n* (%)		
Stage I	43 (47.2)	
Stage II	19 (20.9)	
Stage III	10 (11.0)	–
Stage IV	9 (9.9)	
Stage V	10 (11.0)	
Duration of PD (years)		
Range	1–50	–
Mean (SD)	10.2 (9.4)	
Age at onset of PD (years)		
Range	15–86	–
Mean (SD)	61.7 (13.4)	
Patient had caregiver, *n* (%)		
Yes	85 (93.4)	–
Relation of caregiver with patient, *n* (%)		
Spouse	55 (60.4)	42 (50.6)
Son/daughter	21 (23.1)	27 (32.5)
Resources, *n* (%)		
More than two resources	72 (79.1)	45 (54.2)
Sharing home with patient, *n* (%)		
Yes	–	60 (72.3)
Diagnosed disease, *n* (%)		
Yes	–	48 (57.8)
Comorbidity, *n* (%)		
Yes	66 (72.5)	–
Time as caregiver (years)		
Range	–	1–43
Mean (SD)		6.9 (7.5)

Descriptive statistics on QoL, PAIS-SR, Brief Cope and Benefit finding of the sample are shown in Table 2. Patients and relatives noticed a mild impact on QoL and indicated minor difficulties in the psychosocial adjustment. In relation to coping responses, informal caregivers used coping responses in the Brief Cope more frequently than patients. Finally, both groups of participants recognised more than half of the benefits indicated in the scale favourably.

**Table 2:** Descriptive statistics in the scales

	Patients (*n* = 91)	Caregivers (*n* = 83)
PDQ-39	29.0 (15.4)	–
SQLC	–	104.7 (25.0)
PAIS-SR	36.7 (17.6)	32.5 (15.8)
Brief Cope	53.9 (11.0)	57.8 (10.4)
Benefit finding	41.7 (17.3)	44.2 (17.4)

Results are reported by mean (SD), PDQ-39 (39-item PD Questionnaire), Brief Cope Scale, Benefit Finding Scale

*SQLC* scale of QoL of caregivers, *PAIS-SR* psychosocial adjustment to illness scale self-report

### Factors contributing to psychosocial adjustment and QoL

Correlations among predictors were examined in patients (see Table 3) and relatives (see Table 4) and all bivariate correlations were below 0.70 with the exception of PDQ-39 and PAIS-SR in patients. Also, no collinearity issues were found in the four models because their Variance Inflation Factor (VIF) values were below three. Therefore, all predictors were included in the multiple regression models.

**Table 3:** Correlations between PDQ-39 and predictor variables in patients (*n* = 91)

	PDQ-39	PAIS-SR	Brief Cope	Benefit finding	Resources	H and Y
PDQ-39	–					
PAIS-SR	0.76***	–				
Brief Cope	−0.25*	−0.39***	–			
Benefit finding	0.03	−0.01	0.18	–		
Resources	0.35**	0.27**	−0.02	0.15	–	
H and Y	0.66***	0.48**	−0.04	−0.07	0.43***	–
Patient had caregiver	0.22*	0.25*	0.12	−0.08	0.18	0.13

H and Y (Hoehn and Yahr stage) Pearson correlations, Phi-coefficient, Point bi-serial correlations

* *p* < 0.05; ** *p* < 0.01; *** *p* < 0.001

**Table 4:** Correlations between SQLC and predictor variables in relatives (*n* = 83)

	SQLC	PAIS-SR	Brief Cope	Benefit finding	Resources	Time as caregiver
SQLC	–					
PAIS-SR	−0.59***	–				
Brief Cope	0.01	−0.36**	–			
Benefit finding	−0.38***	0.30**	0.05	–		
Resources	−0.39**	0.44***	−0.24*	0.32**	–	
Time as caregiver	−0.08	−0.02	0.07	0.14	−0.01	–
Sharing home with patient	0.11	−0.01	−0.21	−0.02	0.10	0.07

Pearson correlations, Phi-coefficient, Point bi-serial correlations

* *p* < 0.05; ** *p* < 0.01; *** *p* < 0.001

The multiple regression analyses to evaluate which factors influenced the psychosocial adjustment to PD in patients and informal caregivers are presented in Table 5.

**Table 5:** Multiple regression models of factors influencing psychosocial adjustment to PD in patients (*n* = 91) and caregivers (*n* = 83)

	PAIS patients		PAIS caregivers	
	*B* (95 % CI)	*p*	*B* (95 % CI)	*p*
Adjusted *R* ^2^	0.421		0.271	
Age	−0.36 (−0.67, −0.05)	0.025*	0.17 (−0.05, 0.38)	0.127
Gender	1.69 (−4.12, 7.50)	0.564	1.62 (−5.65, 8.90)	0.658
Brief Cope	−0.61 (−0.86, −0.36)	<0.001*	−0.51 (−0.80, −0.22)	0.001*
Benefit Finding	ns		0.23 (0.05, 0.40)	0.011*
H and Y	18.38 (12.06, 24.70)	<0.001*	–	–
Patient had caregiver	15.65 (5.29, 26.01)	0.004*	–	–
Resources	ns		17.92 (4.92,30.92)	0.008*
Time as caregiver	–		ns	
Sharing home with patient	–		−0.01 (−0.01, 0.00)	0.051

*B* (regression coefficient unstandardized), 95 % CI (confidence interval)

*ns* not statistically significant, *H and Y* Hoehn and Yahr stage

* *p* < 0.05; ** *p* < 0.01; *** *p* < 0.001

The first model (PAIS-SR Patients) explained 42 % (MSE = 13.396) of the variation of the patient’s psychosocial adjustment. The increase in age predicted a reduction in the difficulties for positive psychosocial adjustment. The presence of a caregiver was a determinant for a more difficult psychosocial adjustment. Patient’s coping responses and the Hoehn and Yahr stage were the major determinants of psychosocial adjustment, with higher stages in the Hoehn and Yahr scale predicting a worse psychosocial adjustment. However, coping responses were predictors of an improvement in this. The resources and benefit finding variables were eliminated because they were not statistically significant.

The second model (PAIS-SR Caregivers) explained 27 % (MSE = 13.468) of variability in caregivers’ psychosocial adjustment. Age, gender and sharing a home with a patient did not significantly predict caregivers’ psychosocial adjustment. Finding benefit in the experience was a determinant of a worse psychosocial adjustment. Moreover, the use of many resources was related to greater difficulties in psychosocial adjustment. Finally, coping responses was the major predictor of improvement in psychosocial adjustment. The number of years as an informal caregiver was eliminated because it was not statistically significant.

The multiple regression analyses to determine which factors influenced the QoL in patients and informal caregivers are presented in Table 6.

**Table 6:** Multiple regression models of factors influencing QoL in patients (*n* = 91) and caregivers (*n* = 83)

	PDQ-39 patients		SQLC caregivers	
	*B* (95 % CI)	*p*	*B* (95 % CI)	*p*
Adjusted *R* ^2^	0.660		0.414	
Age	0.15 (−0.08, 0.39)	0.201	−0.12 (−0.44, 0.19)	0.438
Gender	5.24 (1.29, 9.18)	0.01*	−6.90 (−17.29, 3.48)	0.189
Brief Cope	−0.03 (−0.22, 0.16)	0.77	−0.46 (−0.92, −0.01)	0.048*
Benefit finding	0.70 (−0.05, 0.19)	0.26	−0.33 (−0.58, −0.07)	0.014*
H and Y	9.41 (4.35, 14.47)	<0.001***	–	–
Patient had caregiver	−1.61 (−9.14,5.90)	0.67	–	–
Resources	1.17 (−3.93, 6.28)	0.64	−7.35 (−27.76,12.06)	0.453
PAIS-SR	0.54 (0.39, 0.68)	<0.001***	−0.86 (−1.18, −0.53)	<0.001***
Time as caregiver	–	–	ns	
Sharing home with patient	–	–	0.01 (−0.01, 0.01)	0.238

*B* (regression coefficient unstandardized), 95 % CI (confidence interval)

*ns* not statistically significant, *H and Y* Hoehn and Yahr stage

* *p* < 0.05; ** *p* < 0.01; *** *p* < 0.001

The third model (PDQ-39 Patients) explained 66 % (MSE = 9.003) of variability in the patient’s QoL. The severity of the disease (Hoehn and Yahr stage) was the major predictor of patient’s QoL. Patients with higher Hoehn and Yahr stages perceived worse QoL. Additionally, psychosocial adjustment and gender demonstrated statistical significance in relation to QoL. The improvement in psychosocial adjustment was a determinant of better QoL, whereas males predicted worse QoL than females. Age, coping responses, benefit finding, the availability of a caregiver and resources did not significantly predict the patient’s QoL.

The fourth model (SQLC Caregivers) explained 41 % (MSE = 19.174) of variability in caregivers’ QoL. In this model, age, gender, resources and sharing a home with the patient did not significantly predict caregivers’ QoL. The results suggest caregiver’s psychosocial adjustment as the main predictor of their QoL. The increase in difficulties to psychosocial adjustment was related to worse QoL. Perceiving benefit finding in the disease was also a predictor of worse QoL. On the contrary, using various coping responses predicted an improvement in relative’s QoL. The number of years spent playing an informal caregiver role was eliminated because it was not statistically significant.

## Discussion

In the present study, the severity of the disease turned out to be a significant predictor of psychosocial adjustment in PD patients. This result has been demonstrated in a previous study with PD patients [38]. However, other attitudinal and socio-demographic factors have been identified. In our study, coping responses, age and the availability of a caregiver were also found to be significant predictors of psychosocial adjustment in PD patients. A higher number of coping responses determined a better psychosocial adjustment in patients, which may be due to the fact that these skills help to face the stressors associated with the illness [4, 8–10]. The contribution of coping responses in psychosocial adjustment has already been demonstrated in patients with other chronic illnesses [4, 8–10] but it has not been previously studied in PD patients and caregivers. In this study, the importance of coping responses in promoting a positive psychosocial adjustment was found to be consistent in patients with PD and their caregivers. The role of age in patients could be explained by the fact that older patients had lower social responsibilities and duties and they therefore perceived less difficulty in adjusting their life goals to the changes imposed by their illness [4, 39]. Finally, the relationship between having a caregiver and perceiving greater difficulties to psychosocial adjustment may be explained by the fact that patients who had a caregiver were those who had more limitations in their daily lives. However, this result has to be considered carefully because in this study, nearly all of the patients (93.4 %) had a caregiver. Therefore, taking these results into account we consider that patients with higher Hoehn and Yahr stage, lower number of coping responses and young age have greater risk of not achieving a positive psychosocial adjustment to PD.

Regarding predictors of caregivers’ psychosocial adjustment, benefit finding and resources contributed towards having difficulties with this, whereas a higher number of coping responses predicted a better psychosocial adjustment. Previous research into chronic illness has found contrasting results about how much finding benefits in an illness may contribute to the psychosocial adjustment of caregivers and patients [4, 8, 19]. Some studies have identified that positive benefits were reported more frequently by participants after a long time of their stressful event [40]. This is consistent with the results of this study because the diagnosis of PD in participants was about 10 years ago. At the same time, the perception of positive benefits has been associated with the coexistence of stress [40]. This finding was supported in this study because caregivers who perceived positive benefits reported difficulties in the psychosocial adjustment. According to previous research, finding benefit in the illness may help caregivers to make a positive reappraisal of the stressful event in relation to goals and values for coping with the ongoing stress [40]. Hence, we suggest that finding benefits in the illness might be a defense mechanism used by caregivers in this study to face the difficulties of the psychosocial adjustment to PD. Thus, healthcare professionals could facilitate informal caregivers identify possible benefits experienced due to the PD through a therapeutic communication [17, 40]. The relationship between poor adjustment and a higher number of resources may reflect that caregivers who used greater resources were those whose patients required more help to carry out activities of daily living. Consequently, based on these results, caregivers with a lower number of coping responses, higher number of perceived benefits in the illness and higher use of resources are proposed as a group at greater risk of poor psychosocial adjustment.

It is interesting to highlight that in this study, the only predictor of positive psychosocial adjustment in PD patients and caregivers was coping responses, a factor which could be addressed in psychoeducational interventions [40]. Nevertheless, more inquiry about the influence of coping responses in other contexts, taking into consideration other possible factors, such as culture, personality or education, is necessary.

In relation to multiple regression models of QoL, it can be noted that age was not identified as a predictor neither in patients nor in caregivers. This finding is consistent with prior research [14]. In addition, male PD patients predicted worse QoL than females and this may indicate that men perceived a greater impact on their lives due to the physical difficulties, such as impaired mobility, that PD causes [41]. This outcome is consonant with preceding research that states elderly male people show more difficulties to assume their loss of physical capacity than females [41]. This finding may be attributed to the traditional differences in roles [41]. Notwithstanding, future qualitative research would be necessary to explain this result. Using more coping responses predicted better QoL in caregivers but not in patients. This result suggests that coping responses might help caregivers to endure the challenges that impair their QoL [40]. Future research to determine specific coping responses which might improve caregivers’ QoL is encouraged. In this study, finding benefits as a consequence of PD predicted worse QoL in caregivers. As stated before, we suggest that finding benefits could be a defense mechanism for caregivers to deal with stress situations that involves their QoL. In consequence, there is a need for further investigation into the kinds of benefits which influence positively in caregivers’ QoL and psychosocial adjustment to promote it with therapeutic communication. According to prior research we found that disease severity was a strong predictor of QoL in patients [13–15]. Nevertheless, surprisingly the social variables measured in this study (having resources, the availability of a caregiver, time as a caregiver and sharing a home with the patient) did not predict patients’ and caregivers’ QoL. This unexpected finding might indicate that these social factors were less important to participants because they were usual in their context [10]. Finally, it must be emphasized that psychosocial adjustment was a statistically significant predictor of QoL in patients and caregivers. This result has been previously confirmed in Japanese PD patients [10] but it is the first time it has been identified in caregivers. It may be explained by the fact that in PD, personal attitudes developed by patients and caregivers in their day-to-day acquire the same relevance as their physical affectation or clinical symptoms [12, 14, 15, 42]. In fact, there is a great difference in how a person perceives a disease if instead of a temporal acute process, it is a long-term condition. Living with a chronic illness has an impact even in the perception of the self of each person, making necessary for him/her to integrate the illness in a new self [43]. Furthermore the Psychosocial adjustment process helps patients and caregivers to fit their social activities and expectations with their new situation [8]. Therefore, this adaptation process might facilitate the reconstruction of their self-identity and their sense of control in the new situation that seems to affect their QoL.

This result suggests that it may be possible to enhance the QoL of caregivers and PD patients, empowering them towards a positive psychosocial adjustment to PD [11]. The results reported in this study suggest that fostering coping responses is a potential approach, which may help patients and caregivers to improve their psychosocial adjustment. Research into this area is encouraged in order to explore effective interventions to improve coping responses and psychosocial adjustment in PD patients and caregivers. We consider that multidisciplinary interventions which implement peer group sessions and integrate the direction of individual needs with telehealth services could be a potential strategy to reach this aim [44]. According to the results of this study, male patients with advanced Hoehn and Yahr stage and poor psychosocial adjustment could be patients more vulnerable to have worse QoL. Also, relatives who find positive benefits in the illness, use lower number of coping responses, and have difficulties in the psychosocial adjustment might perceive a greater impact in their QoL. In this sense, this group of PD patients and caregivers should receive a special priority in these interventions.

The present study has demonstrated that caregivers of patients with PD in its early stages also had a mild impact on QoL and minor difficulties in the psychosocial adjustment, which were very similar to those in patients. It is therefore necessary for health care professionals to consider the influence that PD may have on caregivers’ QoL and health [45].

The cross-sectional design may be considered a limitation in the present study because it could interfere with proving the cause and effect relationships that have been identified in this research. Therefore, developing longitudinal studies with repeated measures to establish the results of the study is recommended. Secondly, two scales (Benefit Finding and PAIS-SR version for caregivers) were not validated in Spanish at the time of the study. However, a rigorous process of back translation was developed in order to obtain a Spanish version [33]. Thirdly, descriptive characteristics of the sample might have influenced the results. In the present study nearly half of the patients had Hoehn and Yahr stage I; most patients had comorbidity and caregivers; patients with dementia were excluded; and more than half of the caregivers had a diagnosed disease. Further research to explore which is the influence of psychosocial adjustment, coping and QoL in patients and caregivers without these characteristics is necessary. Nevertheless, the prevalence of PD patients with descriptive characteristics reported in this study (see Table 1) is high [1]. In this way, the target population of these results entails a large number of individuals with PD and informal caregivers. Lastly, findings could be influenced by the presence of the researcher in the data collection. However, the participants did not know the researchers.

To sum up, the results supported our hypothesis about the significant influence of coping responses, benefit finding and severity of the disease on psychosocial adjustment. These findings also determine the psychosocial adjustment as a significant predictor of QoL in PD patients and caregivers.

In conclusion, we consider coping responses and psychosocial adjustment to be key personal and attitudinal factors for achieving better outcomes in the QoL of PD patients and their caregivers. Reinforcing these factors, along with the traditional control of symptoms, could result in more effective interventions to manage the neurodegenerative character of PD in patients. It could also prevent negative consequences in informal caregivers, who in these cases are clear examples of long term support for the patient. Consequently, a multidisciplinary approach is suggested in order to deliver comprehensive and person -centered care for PD patients and their informal caregivers [44].
